# EXTraction of EMR numerical data: an efficient and generalizable tool to EXTEND clinical research

**DOI:** 10.1186/s12911-019-0970-1

**Published:** 2019-11-15

**Authors:** Tianrun Cai, Luwan Zhang, Nicole Yang, Kanako K. Kumamaru, Frank J. Rybicki, Tianxi Cai, Katherine P. Liao

**Affiliations:** 1 Division of Rheumatology, Immunology, and Allergy, Brigham and Women’s Hospital, Boston, MA, 6016BB, 60 Fenwood Road, Boston, 02115 USA; 2 Harvard Medical School, Boston, MA USA; 3 Department of Radiology, School of Medicine, Juntendo University, Tokyo, Japan; 4 Department of Radiology, University of Ottawa, Ottawa, Canada; 5 Harvard T.H. Chan School of Public Health, Boston, MA USA; 6 VA Boston Healthcare System, Boston, MA USA

**Keywords:** Natural language processing, Data mining, EMR, Numerical data, Big data, Data extraction

## Abstract

**Background:**

Electronic medical records (EMR) contain numerical data important for clinical outcomes research, such as vital signs and cardiac ejection fractions (EF), which tend to be embedded in narrative clinical notes. In current practice, this data is often manually extracted for use in research studies. However, due to the large volume of notes in datasets, manually extracting numerical data often becomes infeasible. The objective of this study is to develop and validate a natural language processing (NLP) tool that can efficiently extract numerical clinical data from narrative notes.

**Results:**

To validate the accuracy of the tool EXTraction of EMR Numerical Data (EXTEND), we developed a reference standard by manually extracting vital signs from 285 notes, EF values from 300 notes, glycated hemoglobin (HbA1C), and serum creatinine from 890 notes. For each parameter of interest, we calculated the sensitivity, specificity, positive predictive value (PPV), negative predictive value (NPV) and F_1_ score of EXTEND using two metrics.

(1) completion of data extraction, and (2) accuracy of data extraction compared to the actual values in the note verified by chart review. At the note level, extraction by EXTEND was considered correct only if it accurately detected and extracted all values of interest in a note.

Using manually-annotated labels as the gold standard, the note-level accuracy of EXTEND in capturing the numerical vital sign values, EF, HbA1C and creatinine ranged from 0.88 to 0.95 for sensitivity, 0.95 to 1.0 for specificity, 0.95 to 1.0 for PPV, 0.89 to 0.99 for NPV, and 0.92 to 0.96 in F_1_ scores. Compared to the actual value level, the sensitivity, PPV, and F_1_ score of EXTEND ranged from 0.91 to 0.95, 0.95 to 1.0 and 0.95 to 0.96.

**Conclusions:**

EXTEND is an efficient, flexible tool that uses knowledge-based rules to extract clinical numerical parameters with high accuracy. By increasing dictionary terms and developing new rules, the usage of EXTEND can easily be expanded to extract additional numerical data important in clinical outcomes research.

## Background

Electronic medical records (EMR) provide a rich source of numerical data for clinical outcomes research, including vital signs, laboratory values, and physiologic values such as vital signs (VS) and ejection fraction (EF) readings. However, much of this information is frequently embedded in unstructured narrative text. Furthermore, this numerical data often represents a distinct subset of information, usually consisting of a medical term coupled with a numerical value (or range) and with or without a unit or condition followed, and habitually recorded with implied units or conditions, e.g., “Blood Pressure 132/87,” “Saturation 97% Room Air,” “Ejection Fraction 35-55%.”

Existing tools for extracting clinical information from narrative text largely rely on a specific type of value or a specific type of note. For example, MedLEE (Medical Language Extraction and Encoding System), developed by Friedman et al., was designed to extract medical concept information from radiology reports and discharge summaries [1]. Similarly, MedEx, developed by Xu et al. [2], focuses on extracting medication dosing information from discharge summaries. For the growing number of clinical research studies using EMR data, there is an unmet need for a general approach that can efficiently extract numeric data from narrative text. Natural language processing (NLP) systems have been developed to address certain aspects of extracting numeric data from narrative text. However, while generalizable tools such as Mayo clinical Text Analysis and Knowledge Extraction System (cTAKES) [3] and MetaMap [4] can be used to identify medical concepts from text, customizing these tools to perform numerical data extraction is not a simple process.

Additional natural language processing (NLP) systems have been developed by other research teams, with impressive performance in particular clinical subdomains. Torii et al. performed concept extraction using machine-learning taggers [5], while Garvin et al. and Xie et al. employed automatic extraction of EF values from echocardiogram reports with good accuracy [6, 7]. Additionally, Chinmoy Nath et al. developed the NLP tool EchoInfer to extract multiple data elements relating to cardiovascular structure and function (including EF) from echocardiography reports [8]. While NLP tools for extracting numerical data have been developed for additional clinical attributes including vital signs, EF, and disease severity scoring scales, none of these tools have been reported to function as a generalizable tool adaptable to a broad range of numerical data.

Unstructured data often contains key data required for a clinical study. Thus, a general approach or tool that can efficiently extract a broad range of numeric human physiologic data, e.g. blood pressure, EF, and laboratory values, from narrative notes would be directly translatable to studies [9].

The objective of this study was to develop and validate a simple and powerful NLP tool, EXTraction of EMR Numerical Data (EXTEND), which could be used to extract a broad variety of numerical physiologic data across different types of notes, without the need for sophisticated linguistic analysis. We specifically validated EXTEND for extracting VS, EF, and some additional laboratory results, including glycated hemoglobin (HbA1C) and serum creatinine (Creat) levels. From this work, we hypothesize that a simple rule-based approach can be used to design a tool that can efficiently and accurately extract numerical information for a wide range of clinical outcome studies.

### Implementation

**Fig. 1:**
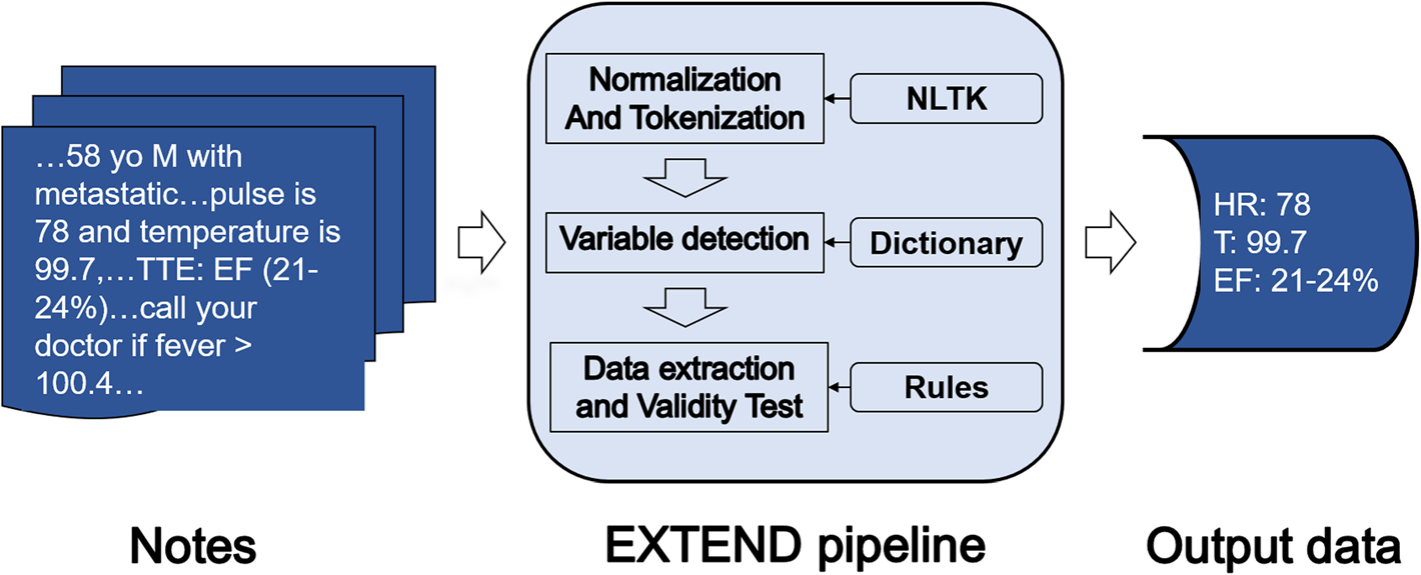
Overview of EXTEND workflow (NLTK: The Natural Language Toolkit)

The proposed tool EXTEND employs a generalizable, rule-based approach. The workflow (Fig. 1**)** of EXTEND consists of three main steps: (i) normalization and tokenization of the notes; (ii) detection of clinical terms associated with the variable of interest; (iii) data extraction and validity tests.

### Normalization and tokenization

**Fig. 2:**
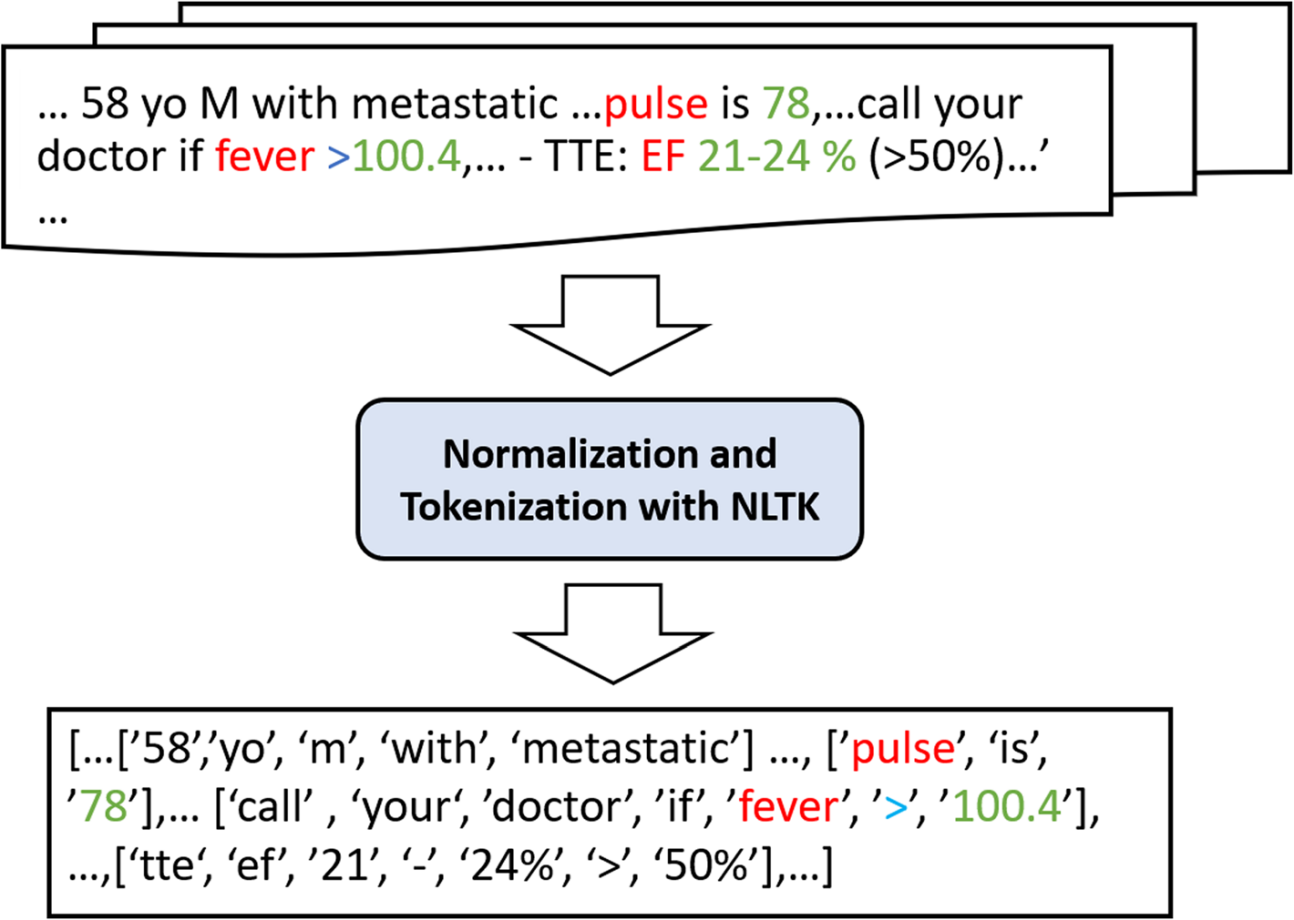
Report Normalization and Tokenization Using the Natural Language Toolkit

After a note is loaded, the first step involves report normalization, followed by sentence and word tokenization (Fig. 2**)**. The Natural Language Toolkit (NLTK) [10], a Python package for NLP, is used to normalize the narrative text, including the removal of grammatical punctuation and conversion of the report string to lowercase. Each report typically contains many non-informative words (i.e., “a,” or “the,” also known as stop words) necessitating increased computing time and memory requirements. However, as some abbreviations in the default stop word list are necessary for identifying numerical data, we customized the NLTK default stop word list to exclude terms appearing in variable dictionaries, a process which will be described later. For example, the abbreviation “T” is on the stop word list, however “T” can also represent “temperature” in this study. The NLTK functions by segmenting the narrative text into sentences. Each sentence was then parsed to create a word list. If a clinical term appeared in any word list, the potential value would also be searched within that word list.

### Detection of relevant clinical terms

To detect the variables of interest in notes, we examined components typically reported in vital signs: blood pressure (BP), heart rate (HR), respiratory rate (RR), temperature (T), and oxygen saturation (O_2_Sat). For each variable, we developed a dictionary by querying the Unified Medical Language System (UMLS) database [11] to retrieve all forms of synonyms, acronyms, and abbreviations. We then augmented this dictionary, including additional abbreviations and clinical terms not listed in the UMLS database, such as “TT,” “T,” and “fever” for temperature. These additional terms, although not synonyms of the target concepts, are frequently used in clinical notes together with the numerical values of interest, thereby necessitating their inclusion. For example, a temperature of 101.2 may be documented as “fever, 101.2”.

### Data extraction and validity test

Once the dictionary is created, EXTEND extracts data by processing notes against the word arrays using a prefix tree to identify terms listed, e.g., “temp” and “fever” for temperature. EXTEND then identifies numerical values adjacent to those terms within the same sentence or word list. EXTEND not only extracts the closest value but can also extract a range. For example, in a sentence “Echocardiogram showed an ejection fraction of 10-15 percent”, the range “10–15%” will be extracted. Once identified, the note is annotated with the designation ‘term – value’ once each pair passes internal validity.

Typically, the presentation of numerical data for vital signs falls into one of two main categories: either a numerical value listed adjacent to the term (e.g., BP 132/78), or a group of numbers with separators. In the first category, a numerical value will either follow or precede the keyword. Most numerical data belong to the first category, including EF, HbA1C and Creat values. To account for both options, we searched for terms and numerical value pairs appearing close to one another, and assigned the value to a variable if the value was deemed valid. For example, in Fig. 2, the value “100.4” is extracted as temperature because it is preceded by the word “fever,” which maps to the concept of temperature in our dictionary.

**Fig. 3:**
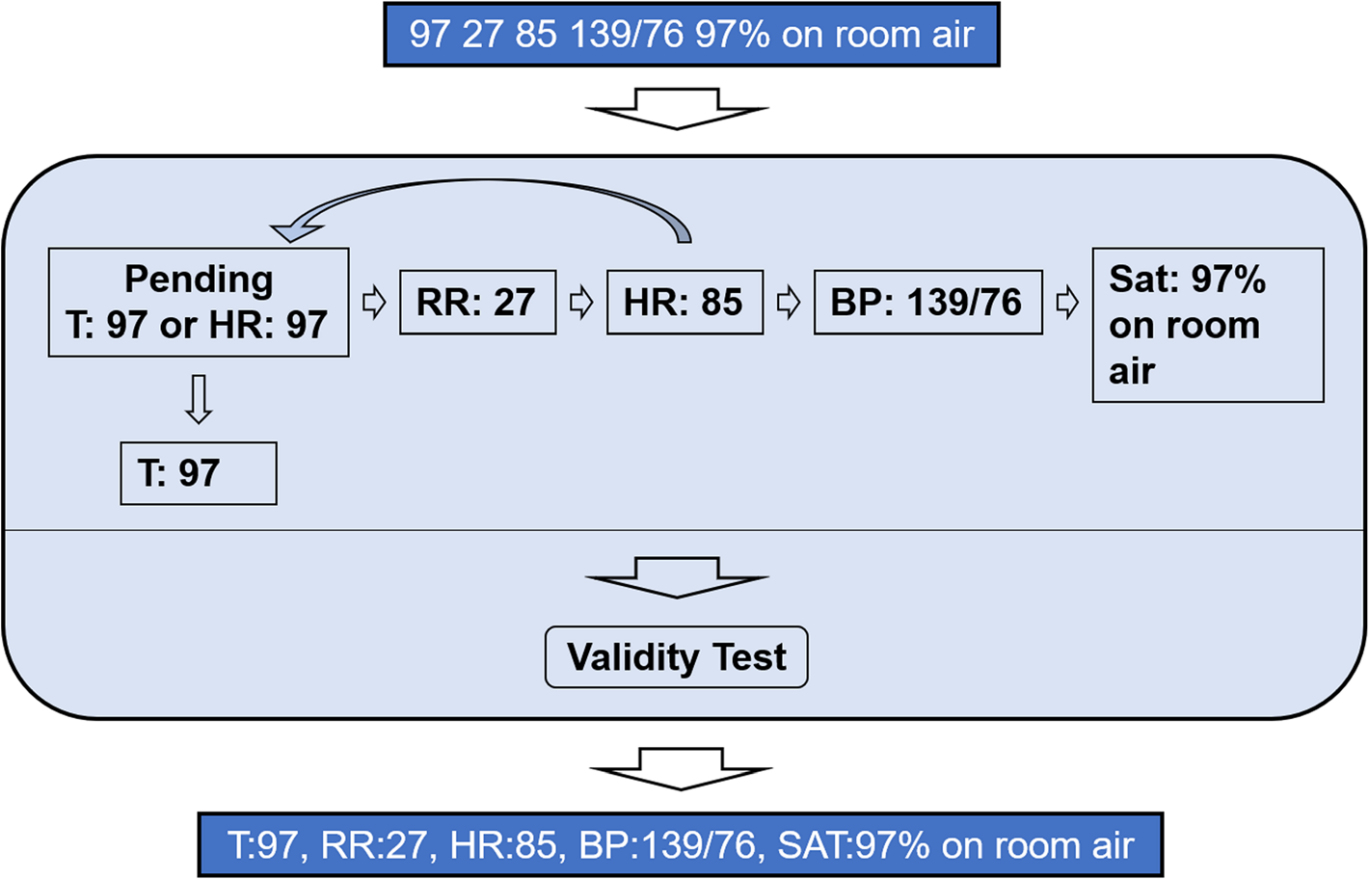
Extraction of a group of vital sign values

In the second category, vital signs (among other variables) may be presented as a group of numbers with separators, such as spaces, without any preceding terms, e.g., “97.0 100/66 98 18 98%”. As observed from chart review, this type of data typically consists of three or more numerical values, and often describes vital sign measurements. Before processing a group of numbers, EXTEND will determine whether they are vital signs (instead of another variable type, such as laboratory test results existing in pseudo-table form) [12] by checking each value against a set of rules. Some examples of the rules assessed include: 1) only containing “.”, “%” and “/” in the number group; 2) at most one “/” or “%” and at most two “.’ in the number group; 3) all integer values less than 300; 4) all decimal values smaller than 110 or larger than 93. Each group of numbers that successfully passes these rules will be considered as potential vital sign measurements, and processed linearly to a relevant variable, identified using an existing set of rules for variable assignment. For instance, in the example given above, the value “97.0” would be assigned to the variable “temperature,” as this represents a decimal number that falls within a predefined range of temperature values for electronic thermometers used in clinical practice and previously entered into EXTEND. Our team employed similar variable assignment rules and predefined ranges for additional vital signs, including, “100/66,” “98,” “18,” and “98%” for BP, HR, RR, and O_2_Sat, respectively (Fig. 3).

Once a variable and value pair has been identified, it is then passed through a validity test using a set of Python functions to incorporate validity test rules. Key information used in each validity test includes a viable range specified by the user, along with the presence of a decimal point, percent symbol, or additional words or symbols indicating a condition, such as “<” and “less than”. For example, the extracted pair “T: 100.4” in Fig. 2 would not be considered a valid pair because “>” lies between the word “fever” and the numerical value “100.4”, suggesting a condition instead of measured temperature data. However, for the extraction of EF or HbA1C, “>” or “higher than” would be included in the expression of measurement, as these represent clinically-valuable ranges in themselves. In addition, unit information is incorporated in each validity test rule to improve disambiguation. If the unit is omitted, the variable can still be determined based on a viable unit range provided for the validity test. For example, in Fig. 1, “99.7” would be assigned to degrees Fahrenheit based on the viable range of human body temperatures.

After testing EXTEND on the extraction of vital signs, a similar methodology was applied to EF data in order to verify overall adaptivity. In similar fashion, we began by expanding our existing dictionary to include EF terms, such as “ejection fraction” and “LVEF,” followed by new rules to assess validity. For instance, the viable range of numerical EF and LVEF was coded to include values lower than 100 (in percent form) or 1.0 in decimal format. For most variables, the general format of value strings could be a decimal number or integer, with or without “%.” To further examine the adaptivity of EXTEND for extracting other types of numerical data, we extracted glycated hemoglobin (HbA1C) and Creat values from EMR, following the steps above with corresponding dictionaries (Table 1) and rules for data extraction and validity testing. As above, these rules included the viable range and possible format of noted values. We observed sufficient accuracy when using the range “< 30%” for HbA1C and “< 50” for Creat.

**Table 1:** Dictionary terms used in EXTEND to search for relevant variables developed manually to identify the common terms used to report these variables in our institution

Variables	Terms
Temperature	t, fever, t-max, fevers, ta, te, tmax, tear, temp, tm, tmp, tmt, tp, tpr, tr, tre, tt, temperature, afebrile
Blood Pressure	b/p, bps, bp, blood pressure, hypertensive, hypotensive
Respiratory Rate	rr, rp, r, resp., respiratory, respiration, respirations, tachypea, breathing
Heart Rate	hr, hrt, p, afib, af, tach, nsr, tachy, pulse, pulses, tachycardia, tachycardic, bradycardic, sinus
Oxygen Saturation	sat, sats, sating, satting, desat, o2sat, o2sats, pox, spo2, sa, sao2, s, oximetry, o2, saturation, saturating, saturations, desaturation, desaturations, desaturates, desaturate, desaturated
Ejection Fraction	ef, ejection fraction, lvef
Glycated Haemoglobin	glycated haemoglobin, glycated hemoglobins, glycated hemoglobin, glycohemoglobin a, glycosylate haemoglobin, glycosylate hemoglobin, glycosylated haemoglobin a, glycosylated haemoglobin, glycosylated hb, glycosylated hemoglobin a’,'glycosylated hemoglobins, glycosylated hemoglobin, haemoglobin a1c, hb a1a + b, hb a1c, hb a1, hba1c, hba1, hemoglobin a1c, hemoglobin glycated, a1c, a1cs, hgba1c, hb1c, hga1c
Creatine	creat, crn, cr, creatinine,scr, cri,creatinin, ctn, cre, crea
Height	h, hgt, hh, ht., height
Weight	wt, w, wgt, wi, bw, weight

### Performance assessment

A variety of notes were utilized for VS and EF extraction, representing documentation typically included in EMR, such as progress reports, discharge summaries, clinic notes, encounter summaries, radiology reports, operative notes, and echocardiogram reports. To assess the performance flexibility of EXTEND, the next phase of our study evaluated the accuracy of data extraction for VS, EF, HbA1C, and Creat across a comprehensive set of medical records, comparing our results against the gold-standard of annotations obtained from manual chart review. For vital sign data extraction, we randomly selected 285 notes from patients in a study of mortality after a diagnosis of pulmonary embolism [9]. For EF extraction, 300 random notes were selected from patients potentially having heart failure. For HbA1C and Creat, 890 random notes were selected from 202 patients at high risk of type 2 diabetes.

We assessed performance characteristics on two levels: the note level and the value level. Using EF as an example, true positive cases occurred at the note level if EXTEND identified the same number for EF in a note as a chart reviewer. False positive cases occurred if the number of correct EF values identified through EXTEND was higher than the number of correct EF values identified through chart review, while false negatives would occur if EXTEND extracted fewer correct values than the true number of values available in the notes. True negatives were cases where EXTEND detected no EF in the notes and the chart reviewer also detected no EF values in the notes.

On the value level, EXTEND was evaluated using the following schema. True positive cases represented those where the EXTEND values matched the values from chart review. False positive cases were noted where EXTEND provided a value where none was present (e.g., where EXTEND provides an EF 45% when there is no EF value in the note). Similarly, a false negative case would occur if EXTEND did not provide a value when a value was provided in the notes. In assessing the accuracy of the value, a true negative could not be calculated, since by definition, no value existed in the notes for comparison. Thus, the performance characteristics used for evaluating accuracy at the value level were sensitivity (also called recall), PPV (also called precision) and F1 score, since NPV cannot be calculated without a true negative.

On the note level, we reported the mean sensitivity, specificity, positive predictive value (PPV), negative predictive value (NPV), F_1_ score and 95% confidence interval (CI) using the bootstrap method to resample 1000 times with replacement. On the value level, sensitivity, PPV, F1 score and 95% confidence interval (CI) were assessed.

## Results

The speed of extraction for EF was observed to be 6260 notes per minute, across all clinical note types, on a desktop with 4-core CPU and 4 GB computer memory. The tool could be further programmed to perform multithreading if computing infrastructure allows.

The performance of EXTEND was evaluated on both the note level and the value level using a reference standard of a total of 1475 unique notes: 285 for VS extraction, 300 for EF, 890 for HbA1c and Creat. At the note level, the performance of EXTEND across the different clinical parameters (BP, HR, RR, T, O2sat, EF, HbA1C and Creat) ranged from 0.88 to 0.95 for sensitivity, 0.95 to 1.0 for specificity, 0.97 to 1.0 for PPV, 0.89 to 0.96 for NPV and with F1 scores ranging from 0.92 to 0.96 (Table 2). At the value level, when evaluated based on the correct values, the sensitivity ranged from 0.91 to 0.95, and PPV ranged from 0.95 to 1.0. The F1 score was 0.95 to 0.96 across the different types of clinical parameters extracted (Table 3).

**Table 2:** The performance of EXTEND to identify vital signs and EF on note level

Clinical parameters	Sensitivity	Specificity	PPV	NPV	F1-score
HR	0.95 (0.92, 0.99)	0.95 (0.92,0.99)	0.97 (0.94,1.0)	0.93 (0.88,0.98)	0.96 (0.94,0.98)
BP	0.91 (0.87, 0.96)	1.0	1.0	0.89 (0.83,0.94)	0.95 (0.93,0.98)
T	0.94 (0.90, 0.98)	0.99 (0.98,1.0)	0.99 (0.97,1.0)	0.96 (0.93,0.99)	0.96 (0.94,0.99)
RR	0.94 (0.91, 0.98)	0.99 (0.98,1.0)	0.99 (0.97,1.0)	0.96 (0.93,0.98)	0.96 (0.94,0.99)
O2Sat	0.94 (0.90, 0.98)	0.98 (0.95,1.0)	0.99 (0.97,1.0)	0.9 (0.85,0.96)	0.96 (0.94,0.98)
EF	0.88 (0.77, 0.99)	1.0	1.0	0.99 (0.97,1.0)	0.94 (0.87,1.0)
HbA1C	0.91 (0.88, 0.94)	0.98 (0.97,0.99)	0.96 (0.94,0.99)	0.96 (0.94,0.97)	0.94 (0.92,0.96)
Creat	0.88 (0.85, 0.92)	0.98 (0.97,0.99)	0.95 (0.92,0.97)	0.94 (0.93,0.96)	0.92 (0.89,0.94)

Abbreviations: *HR* Heart rate, *BP* Blood pressure, *T* Temperature, *RR* Respiratory rate, *O*_*2*_*SAT* Oxygen saturation, *EF* Ejection fraction, *Hba1c* Hemoglobin A1C, create, creatinine. Brackets indicate 95% confidence interval

**Table 3:** The performance of EXTEND to identify vital signs and EF on value level

Clinical parameters	Sensitivity	PPV	F1_score
HR	0.95(0.92,0.99)	0.97(0.95,1.0)	0.96(0.94,0.99)
BP	0.91(0.87,0.96)	1.0	0.95(0.93,0.98)
T	0.94(0.9,0.98)	0.99(0.97,1.0)	0.96(0.94,0.99)
RR	0.94(0.9,0.98)	0.99(0.97,1.0)	0.96(0.94,0.99)
O2Sat	0.94(0.9,0.98)	0.99(0.97,1.0)	0.96(0.94,0.98)
EF	0.92(0.81,1.0)	1.0	0.96(0.89,1.0)
HbA1C	0.95(0.92,0.98)	0.95(0.92,0.98)	0.95(0.93,0.97)
Creat	0.95(0.92,0.97)	0.97(0.96,0.99)	0.95(0.93,0.97)

Numbers in parentheses denote 95% confidence interval (CI). *HR* Heart rate, *BP* Blood pressure, *T* Temperature, *RR* Respiratory rate, *O*_*2*_*SAT* Oxygen saturation, *PPV* Positive predictive value, *NPV* Negative predictive value. Brackets indicate 95% confidence interval

The dictionary created for BP, HR, RR, T, O2sat, EF, HbA1C and Creat can be found in.

During the initial assessment of the performance for HbA1C extraction, our observed sensitivity was 0.85, much lower than the observed performance of data extraction for VS and EF. Upon further review, we found an abbreviation of HbA1C, “hga1c”, which appeared across the clinical notes we examined had not been included in our initial dictionary. After adding this abbreviation to the dictionary, sensitivity for HbA1C extraction increased to 0.90. As a result, our team suggests performing additional chart review to identify potential nuances in abbreviations or re-examining existing algorithm rules when performance is lower than expected.

## Discussion

We observed that leveraging a rule-based approach allowed for the development of general tool, EXTEND, for identifying and extracting numerical data from a variety of clinical records. Additionally, these data can be extracted in a high-throughput manner. In contrast to existing NLP applications, which focus on extraction from specific note types, e.g. discharge summaries, echocardiology reports, EXTEND was trained on a wide range of note types and did not require incorporating complex linguistic analysis. Non-linguistic laboratory measurement extraction is particularly useful in capturing results for test results reported in narrative notes using data performed at other hospitals. Such a tool can enhance clinical chart reviews from outside hospital records as well as broaden the potential data available for clinical outcomes research.

An earlier version of this tool was applied to 69,406 clinic records, including visit notes, radiology reports, progress reports, and discharge summaries from 1698 patients in the Partners Healthcare system to extract vital signs. These data were used to build a scoring system to predict 30-day mortality after acute pulmonary embolism [9].

In comparison to existing technologies, EXTEND provides the advantage of operating on a generalized framework that can be modified to extract different types of numerical data across a variety of clinic records. For instance, using EXTEND would allow investigators interested in studying EF to include data on patients where EF is mentioned in the clinical notes, but where an echocardiogram report is not available. Indeed, the adaptability of this general approach to data extraction across numerous different types of numerical data - BP, T, RR, O2 sat, EF, HbA1C and Creatinine, and different note types, is a particular strength of EXTEND.

To adapt this tool to perform numerical data extraction beyond those listed in this manuscript, users can 1) query UMLS on a local computer or an online website such as “https://www.allacronyms.com” to obtain the term list for generating a dictionary for a variable of interest; 2) manually review a corpus of number notes to assess whether additional terms should be added to optimize the dictionary; 3) have knowledge of the viable range, possible unit and general format of values of the variable for generating rules of validity test.

## Limitations

When performing the development of the purpose-specific dictionary, we included some additional words and abbreviations as keywords not available in the UMLS such as “TT” and “T”, which were identified by manually reviewing a small number of reports. We also included words such as “fever” that were not abbreviations, synonyms or acronyms of variables, but which were potentially followed by numerical data. These additions can improve performance but may cause overfitting.

For performing the validity test to examine if an extracted value is valid, we provided a viable range for a variable such as 93–110 for temperature for improving the accuracy. It may not be as easy to decide a viable range for some variables such as the lower border of EF. Another limitation of the study was that the tool was validated only in two large academic hospitals. The structure of clinical narratives and the expression of medical concepts are diverse in different hospitals and regions. When applying the tool to a different hospitals, additional tailoring of keywords may be necessary.

## Conclusions

EXTEND is a novel, efficient, flexible tool that can be used to accurately extract numerical data from a variety of clinical EMR narratives when compared with manual chart review. High-throughput extraction of numerical data can provide key information for large scale clinical studies using EMR data. While our examples demonstrate its use in vital signs, EF, and laboratory values, the general methods behind EXTEND allow for expansion to other types of numerical data available in the clinical narrative notes.

### Availability and requirements

**Project name:** EXTEND.

**Project home page**: https://github.com/TianrunCai/EXTEND

**Operating system(s):** Platform independent.

**Programming language**: Python.

**Other requirements**: Python 2.7, NLTK module.

**License:** NO. 25122, Brigham and Women’s Hospital.

**Any restrictions to use by non-academics**: license needed.

## Data Availability

The python pyd files of the pipeline, setup scripts and a readme file are available on: https://github.com/TianrunCai/EXTEND

## Abbreviations

BP: Blood pressure
Creat: Serum creatinine
EF: Ejection fraction
EMR: Electronic medical record
EXTEND: The Extraction of EMR numerical data
HbA1C: Glycated hemoglobin
HR: Heart rate
NLP: Natural language processing
NLTK: The Natural Language Toolkit
NPV: Negative predictive value
O_2_Sat: Oxygen saturation
PPV: Positive predictive
RR: Respiratory rate
T: Temperature
UMLS: Unified Medical Language System
